# Utilization of Mobile Mental Health Services among Syrian Refugees and Other Vulnerable Arab Populations—A Systematic Review

**DOI:** 10.3390/ijerph17041295

**Published:** 2020-02-18

**Authors:** Adeel Ashfaq, Shawn Esmaili, Mona Najjar, Farva Batool, Tariq Mukatash, Hadeer Akram Al-Ani, Patrick Marius Koga

**Affiliations:** 1 Department of Surgery, Kaiser Permanente Los Angeles Medical Center, Los Angeles, CA 90027, USA; 2 ULYSSES Project University of California, Davis, Davis, CA 95616, USA; ssesmaili@ucdavis.edu (S.E.); mgnajjar@ucdavis.edu (M.N.); fbatool@ucdavis.edu (F.B.); tmukatash@ucdavis.edu (T.M.); haalani@ucdavis.edu (H.A.A.-A.); 3 Department of Public Health Sciences, School of Medicine, University of California, Davis, Davis, CA 95616, USA

**Keywords:** mental health, mobile health, mMHealth, telehealth, refugees, health capacity, Arab Region, Syrian refugee

## Abstract

The global refugee crisis is at its most critical state in history; Syria alone has produced 12 million internally displaced persons, with another 5 million refugees seeking protection across the globe. Faced with the heavy burden of mental distress carried by a massive refugee influx, many host nations lack the service capacity to respond adequately. While mobile mental health (mMHealth) applications and platforms have the potential to augment screenings and interventions for vulnerable populations, an insufficient gender and cultural adaptation of technology may drastically hamper its uptake in Arab refugees. Reporting only papers originating from Middle Eastern and/or Arab nations or refugee host nations, this systematic review evaluates the available literature published between 2000 and 2019 on the usage acceptability of mMHealth in Syrian refugees and other vulnerable Arab populations. We conducted a systematic review in PubMed, PsychInfo, Association of Computing Machinery (ACM) and the Directory of Open Access Journals (DOAJ) using Preferred Reporting Items for Systematic Reviews and Meta-Analyses (PRISMA) guidelines to identify studies that addressed mMHealth implementation in these populations; of a total of 607 articles identified, only 10 (1.6%) available, unique articles met our search criteria. These studies discussed the feasibility and efficacy of mMHealth applications and the barriers to their uptake. The few existing studies show positive impacts of mMHealth on the access to services and on treatment outcomes but also reveal a paucity of literature on mMHealth for vulnerable Arab populations. These findings indicate a critical need for research on the barriers to mMHealth uptake, to bolster service capacity in the Arab Region and in the refugee diaspora of other, non-Arab host countries.

## 1. Introduction

### 1.1. Background

The global refugee crisis is currently at its most critical point it has ever been in history. In the last three decades devastating wars and violent conflicts in the Middle East, Africa, Central America, Eastern Europe and Central Asia have created millions of refugees worldwide, ushering in a new age of globalized forced migration and drastically shifting diasporic landscapes. By the end of 2017, 68.5 million individuals were forcibly displaced worldwide as a result of violence, persecution, conflict and human rights violations [1]. Notably, this statistic reflects 40 million internally displaced individuals, 3.1 million asylum-seekers and a staggering 25.4 million refugees globally—the highest and most significant ever recorded [1,2].

Among the current and ongoing humanitarian crises, the Syrian refugee crisis remains a disaster as Syrians remain the largest forcibly displaced population in the world with over 12 million people displaced (over half of the pre-conflict population). Over 5 million Syrians currently seek refuge in bordering nations including Lebanon, Turkey and Jordan, as well as several European nations, with the vast majority living under the poverty line and many living in exile [2,3,4]. Additionally, this mass exodus and migration has created significant constraints on host-country infrastructure, political resettlement action and the health of 10′s of millions of refugees.

Refugees and immigrants, particularly from war-torn countries and areas of severe displacement, are considered an extremely vulnerable population, thus carry a higher burden and risk of developing mental health disorders [5]. The refugee experience is divided into three broad stages—preflight/premigration, flight/migration and resettlement/postmigration [3]. During the preflight stage, individuals are subject to traumatic experiences both emotionally and physically due to various factors including witnessing/participating in violence, death, social breakdown, abandoning their roots and homes and so forth. The flight stage includes the actual physical journey from one’s home to the destination in which they are seeking refuge. During this travel period, families are sometimes subject to separation, especially children and adolescents and some face violence, discrimination and duress. In the final, resettlement stage, refugees attempt to adapt themselves to their new environment, often facing difficulties such as the loss of culture, community, language, social capital and support systems [3,4]. Additionally, refugees in this stage experience disorientation in an alien landscape and are compounded further with unemployment, poverty, unsafe housing and so forth. [3,4].

The World Health Organization (WHO) estimates that millions of refugees suffer from chronic mental disorders, psychosocial dysfunction and distress and regards the mental health of refugees and immigrants as a top priority of its efforts. [6]. The World Bank has reported that mental illness accounts for 10% of the overall burden of disease worldwide, including death and disability and as such, is one of the major causes of losses in QALYs (Quality-Adjusted Life Year) and DALYs (Disability-Adjusted Life Year). Mental illness impacts not only health but also the social and economic well-being of individuals, families and societies; due to depression and anxiety, the global economy loses about $1 trillion every year in productivity [6]. Pilot studies conducted on sample sets of Syrian refugees report that the prevalence of post-traumatic stress disorder is over 80% and the population is also suffering from widespread depression, rendering Syrian refugees among the world’s most vulnerable populations [7]. Indeed, the significant mental health burden of this population may have catastrophic long-term consequences for Syrian refugees globally.

With such an immense influx of refugees, the limited legal and healthcare infrastructure of host nations may be unable to provide adequate psychosocial integration or healthcare provisions to refugees. This issue is further compounded with conflicts of social acceptance, cultural integration, rejection and violence from host communities, poverty and general lack of Arabic speaking interpreters. As Syrian refugees continue to migrate and settle, there are many issues to consider in the intersection of health, economics and politics of host nations. The displacement, resettlement and drastic changes that refugees face, are synonymous with changes in the stability of their mental health. It is difficult to address such health issues for multiple reasons but it is predominantly due to limited availability of clinicians along with a host of geopolitical, topographical, infrastructural challenges and social stigma that are creating barriers to clinical treatment and/or social acceptance of mental health issues [6].

### 1.2. Study Motivation

Questions remain as to how host nations or other parties can bolster capacity to alleviate the mental health burden of refugees. Given the current challenges to health systems in war-torn and war-impacted Arab countries, telehealth seems to offer a more viable solution than clinic-based direct care. Though many efforts have been put forth, one strategy that has recently gained momentum is the creation of mobile health (mHealth) and mobile mental health (mMHealth) interventions to achieve healthcare objectives and transform healthcare delivery worldwide.

There is no standardized definition of mHealth; however, the WHO’s Global Observatory for electronic Health (GOe) defines mHealth as medical and public health practice supported by mobile and wireless devices [7]. The recent rapid rise in mobile phone penetration in developing countries could be leveraged to mitigate the challenges of a high population growth (whether through natural increase or through immigration), a high burden of disease prevalence, insufficient health care workforce, of large rural and/or marginalized immigrant populations and of inadequate financial resources to support healthcare infrastructure and health information systems [8]. With both providers and consumers having a greater access to mobile phones, the costs of delivering e-healthcare drop. Naturally, mHealth is not without its share of criticism. Disparities in low- and middle-income countries could exacerbate new forms of digital healthcare exclusion [9]. A rapid uptake of mHealth might alter the practice of healthcare and patient-physician relationships with unintended ill consequences if a preparatory technological education and cultural adaptation has not been adequately put in place to ease an organic transition to e-health [10]. In the context of electronic health records, patient confidentiality and data protection are of special concern [11]. Nevertheless, since the benefits of mHealth uptake outweigh the vulnerabilities mentioned above, especially in countries pressured by a sudden massive influx of refugees, one would expect to see strong policies supporting a faster uptake of mMHealth. Given the widespread penetration of mobile phone networks, even in low-and-middle income nations, this idea carries significant feasibility [6].

Pilot studies and initial reviews from other mHealth areas have reported on successes and barriers to mHealth success. A review of health system utilization by Sarria-Santamerra et al. discusses the general lack of utilization of health services by immigrant populations in a variety of host nations and conclude that further studies are needed to evaluate patterns of health resource usage among these populations [12]. A study from Ankara, Turkey identified language as a major barrier for utilization of health services [13]. The Turkish Ministry of Health is now employing Syrian Health Workers to improve access to Syrians living in Turkey [14]. Additionally, another study by Batniji et al. describes political and governmental instability and accountability as major barriers to all health services in the Arab world [15]. This issue is further compounded by the massive influx of Syrian refugees in these countries. Despite significant barriers, there have been pilot successes in mHealth. A study by Saleh et al. analyzed an mHealth platform for non-communicable disease management among Syrian refugees in a Lebanese refugee camp and found a significant decrease in mean systolic and diastolic blood pressures and HbA1c levels [16].

#### Cultural Modulators of Distress and Treatment Acceptance

However, many factors and barriers are left to consider in terms of social acceptability, cultural and gender norms and infrastructure for mMHealth success. Currently, there is a paucity of literature regarding the development and implementation of mMHealth interventions for mental health among refugee populations, especially in predominately Arab nations. The effectiveness of mobile health technologies to produce desired outcomes (e.g., screening for mental distress) may be reduced when used with an application that is not specifically designed for mHealth (e.g., Skype or WhatsApp). Furthermore, when mMHealth applications fail to formulate their questions (e.g., screening questions) in alignment with Arab idioms of distress, the Western mental constructs conveyed by such questions may not be clearly understood by Arab laypersons whose cultural expression of suffering may be different. Lastly but not in the least, the mode in which mobile health application may convey their questions to users may ignore or even conflict with Arab social norms of gendered communication. For example, questions probing a female patient’s sex drive would be seen as unacceptable or even violating family space and sense of honor. For example, in a study conducted in 2011 by Barber et al. with 68 Palestinians in the West Bank, East Jerusalem and the Gaza Strip, the participants articulated their suffering in the following Arabic terminology: broken, crushed (محطمة muḥaṭṭima), shaken up (مهزوزة mahzūza), destroyed, (مدمرة mudammira) and exhausted, tired (تعبانة ta’bāna) [17]. The aspects of the sufferer were described as the self or spirit (النفس an-nafs), morale (المعنويات al-ma’naūiyyat) and hopes or aspirations for the future (أمالك أو طموحك بالنسبة للمستقبل amālak aū ṭamūḥatak ban-nisba lil-mustaqbil) [17].

The Arab construct of “hozon” (حزن, sadness and difficulty in the face of an acute or sudden stressor) may be referred to by Syrian refugees as al-hayat sawda (الحياة السودا ,‘a black life’) or iswadat al dounia fi ouyouni (اسودّت الدنيا في عيوني, ‘life has blackened in my eyes’) or could be used to signal intense grief from losses and withdrawal from social life [17,18]. When intended to signify a chronic state of depression, “hozon” is replaced by laypersons and mental health practitioners alike with halat ikti’ab (حالة اكتئاب, ‘condition of ikti’ab’), an agglutinant concept of brooding, darkening of mood, aches, a gloomy outlook, somatization and social withdrawal [19].

Moreover, many Arab refugees do not perceive their suffering as the “clinical picture” of a mental disorder and reject the pathologization and overmedicalization of their war-induced distress [20]. The meaning they attribute to their extreme grief is that of a commensurate yet natural response of a normal human being who has been forced to live under extreme conditions of duress. This may require at times a de-pathologized naming of human suffering, as Joseba Achotegui argues in his “Ulysses Syndrome” [21].

Not only are the idioms of distress different in the Arab world, the very construction of personhood (embedded versus autonomous) and the relating, positioning and communicating across sexes is vastly different from Western norms [22]. Consequently, for a successful uptake, Western information and communication technology (ICT) transfer, in general and mMHealth in particular, may need to adapt in alignment with the gender and cultural reality of the Arab consumer.

The problem is that historically there appears to be not only a lack of mMHealth technology applied to the Arab and Syrian refugee populations but also a lack of cultural adaptation of the innovation [23]. Many attempts to apply a technology acceptance model (TAM) have ignored the models’ weaknesses in predicting the cultures and behaviors of individuals and organizations within the complex health domain [24]. Such lack of differentiation between technological and human factors limits mMHealth practical applicability [25]. The development of a cultural and gendered innovation in mobile mental health in this region may better reflect the complex interactions between refugees, health systems and technology and may actually yield a higher user uptake [26], This matter has not been dealt with as rigorously as it should have been in the mMHealth literature. We surmise that part of the problem is the lack of an established theory base and of an evidence-based, consistent, gendered and culturally competent conceptual framing and grounding of mMHealth development, diffusion and transfer.

The present systematic review intends to investigate the current literature available regarding mMHealth and to elucidate key findings of outcomes, successes and barriers. Generally, the authors intend to highlight the current academic landscape of the mMHealth concept to some of world’s most vulnerable populations: Arab vulnerable populations and Arab refugees in the Arab region and in non-Arab host countries. Our three pronged systematic review sought to explore the existing research literature on (1) the current presence, uptake and outcomes of mMHealth applications among Arab populations and Arab refugees, (2) the mMHealth usage acceptability and barriers to use among this populations and (3) the mMHealth adaptation to Arab culture and gender norms.

## 2. Materials and Methods

To analyze the existing literature regarding mMHealth applications in Syrian refugees and Arab populations, we conducted a literature review in the following four databases: PubMed/Medline, PsychInfo, Association of Computing Machinery (ACM) and the Directory of Open Access Journals (DOAJ). To ensure validity and standardization of our methods, we utilized the Preferred Reporting Items for Systematic Reviews and Meta-Analyses (PRISMA) guidelines [27]. The context of our research questions is provided in Table 1, utilizing a format provided by PRISMA [27]. This literature review was conducted from March 2019 to November 2019.

### 2.1. Information Source and Search Strategy

The review considered English language quantitative and qualitative primary studies on mMHealth published between 2000 and 2019 in the peer-reviewed literature from four databases: PubMed, PsychInfo, ACM and DOAJ. The retrieved journal articles were downloaded into Endnote X8 citation management software and duplicates were removed. The authors were divided among two primary research teams, each with the same tasks: (1) Screen the four databases with our standard search methodology and (2) Evaluate the quality of the articles. After the primary researchers of each team screened the titles and abstracts, the remaining members of the research team reviewed the results based on the inclusion and exclusion criteria. Two quality appraisal meetings with both teams were held to discuss all uncertainty regarding articles for inclusion or exclusion articles for inclusion. The remaining relevant articles were organized into three major categories—excluded literature, background literature and possibly relevant literature.

We utilized a search algorithm using Boolean methodology to create the ideal search query for all 4 databases. The search algorithm encompassed all possible terminology for mHealth, as well as a series of factors that qualified the search. The exact search queries for each journal are tabulated in Table 2.

### 2.2. Inclusion/Exclusion Criteria

Articles were included only if they (1) originated from Middle Eastern and/or Arab nations or refugee host nations; (2) focused on a study population of Arabic speakers and/or Syrian refugees; and (3) primarily discussed mMHealth applications and/or feasibility of this technology within the study population. The review excluded studies published before 2000, grey literature and non-peer-reviewed articles. Only articles that met all criteria were included for further evaluation. The included articles were assessed for key findings related to mMHealth within the study population of interest.

### 2.3. Data Extraction

**Figure 1:**
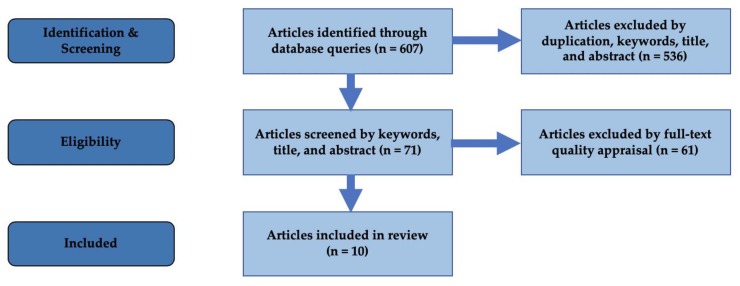
PRISMA flow diagram of review process.

Each research team performed a criteria-based data extraction (citation, participant characteristics, methods, results, key conclusions, recommendations); afterwards the results were cross-checked by two researchers for any possible data errors. Figure 1 shows the PRISMA process undertaken for this review.

### 2.4. Inter-Rater Reliability

The selection phase of this review was rigorously based on inter-rater agreement or disagreement between the two research teams. Cohen’s kappa coefficients (*k*) were calculated to quantify inter-rater agreement. We used the IBM Statistical Package for the Social Sciences (SPSS) version 22.0, IBM, Armonk, NY, USA, to determine the statistical significance of agreement between the research teams. The primary response categories were “Yes” or “No/Not applicable.” “Yes” referred to agreement on selecting an article for inclusion, while “No/Not applicable” meant disagreement on article inclusion. The two primary categories of agreement were “Initial Screening” and “Quality Evaluation.” The category “Initial Screening” was then divided among agreement/disagreements of inclusions based on the following—title, abstract, full article and type of article (reviews, outcomes studies, qualitative studies, randomized control trials, etc.) and additionally the level of comprehensiveness of the search. The category “Quality Evaluation” was then divided among agreements/disagreements regarding quality disputes of the following—topic, study design and quality of the research journal.

## 3. Results

### 3.1. Review Results

A total of 607 articles were identified using the Boolean search queries on PubMed, PsychInfo, ACM and DOAJ databases. Of the 607 articles, only 10 (1.6%) unique articles met all inclusion criteria. Of the 10 included articles, 7 studies discuss the utilization of novel mMHealth applications, 5 discuss barriers, 5 discuss attitudes and acceptance of mMHealth, 1 is a randomized crossover study and 1 is a randomized control trial analyzing efficacy of an mMHealth prototype. Seven unique mMHealth prototypes were discussed and included 3 web-based interventions, 2 mobile applications, 1 video/audio transfer tool and 1 tele-conferencing tool. The articles report 64–88.2% of their study populations express positive interest in mMHealth applications and positive attitudes toward mMHealth utilization. All 7 individual mMHealth technologies were efficacious as screening tools and psychiatric treatment and the majority of participants and providers had positive attitudes and displayed acceptance of mMHealth. Additionally, the studies report 64–93.4% of participants owning a mobile phone and being connected to a mobile network. The randomized control trial shows significant efficacy of a mMHealth application prototype on symptom relief of post-traumatic stress disorder. The randomized crossover trial showed good efficacy and feasibility of a mobile application. Barriers discussed primarily included primarily cultural, financial, technical, infrastructural and data privacy limitations and lack of mental health awareness in the study populations. Results and key findings are tabulated in Table 3 [6,28,29,30,31,32,33,34,35,36].

### 3.2. Inter-rater Reliability

The k calculated for each agreement/disagreement category and sub-divisions were consistently high in the initial screening and quality appraisal phases of this review. These results statistically, indicate consistent agreement among the authors and teams regarding the inclusions/exclusion of studies. The values of Cohen’s Kappa showed an acceptable range of 0.83–0.95. In an overall assessment of inter-rater reliability, the screening and evaluation process showed significant agreements (*p* < 0.001) regarding the selection of articles with the given inclusion and exclusion criteria. These results are tabulated in Table 4.

## 4. Discussion

The few studies that were available discussed implications, efficacy and barriers to mMHealth applications within the target population. Though only seven articles included in this review referred to mobile phone applications, their data clearly suggest that mMHealth applications carry high feasibility and potential, as many patients in the target areas of the Middle East own a mobile phone and have access to a cellular network [12]. Additionally, the cross-sectional studies suggest that the majority of Arab patients and Syrian refugees perceive mMHealth as positive and welcome the prospect of using mobile technology to monitor and bolster their mental health, among other use cases of this novel technology [4,28,29]. In terms of statistical efficacy of mMHealth vs conventional treatments, not many studies were available. However, the single randomized control trial included in the present study found a statistically significant reduction in post-traumatic stress syndrome symptoms in Arab patients from war-torn backgrounds [34]. Additionally, the remainder of the articles showed efficacy of the technologies as screening tools for this population [6,28,29,30,31,32,33,34,35,36]. It is imperative that mMHealth innovations are compatible with local needs of intended adopters and implementing institutions. This is evidenced by the barriers identified in five of the articles including inadequate technological capacity, insufficient or lack of cultural adaptation and acceptance and poor credibility as barriers to mHealth implementation [28,29,31,34,35]. Given the extent of the current refugee crisis and the incidence and prevalence of mental health disorders among these vulnerable populations, it is imperative that further studies are conducted to evaluate the efficacy of this potentially groundbreaking technology.

With regard to the success of mMHealth uptake among vulnerable Arab populations, many barriers exist spanning stigmatization, technological literacy, technological access, general distrust towards healthcare providers and political conflicts [14,28,30,35]. In Arab communities, there is a strong social stigma associated with mental illness as men who seek care are considered weak and women associate it with helplessness and being ‘cursed’ [12,37,38]. Another barrier is the preferred provider-patient relationship in this population, in which Arabs expect an authoritative style of communication where providers give concrete and specific solutions rather than long-term, “collaborative,” dynamic psychotherapy [37].

Additionally, technological barriers can significantly hinder mHealth uptake, including lack of access to Wi-Fi networks. A survey conducted of Palestinians in the West Bank showed an estimated 80% of the population have regular access to a smartphone, with 70% having a pay-as-you-go plan, whereas in Egypt only 45% of the Syrian refugees have access to a smartphone [12]. Another study from Syrian refugees in Egypt found 70% of refugees reported the cost of mobile access to be a barrier [12,37]. Additional barriers reported from the study were—lack of credibility of services (57%), acceptability of the application (47%) and technical literacy (25%) [12].

Mobile mental health is a promising way to reach a large portion of this vulnerable population; since most refugees have smartphones, the accessibility of a mobile app technology is feasible for usage. Additionally, mental health services in general that have been culturally, religiously and linguistically adapted to refugees in the Levantine region (Syria, Lebanon, Palestine) have been met with overwhelmingly positive results [13,15,16,37]. Therefore, the ideal mMHealth platform would incorporate linguistic, cultural and religious adaptations.

mHealth is a fairly novel concept, thus the literature is generally lacking in evidence of success globally and in all forms of healthcare from mental health to communicable diseases to trauma systems registries. Despite its novelty, mHealth has had some inspiring successes that may serve as precedents for governments and NGOs around the globe. A review article by Abaza and Marschollek published in 2017 identified 255 distinct articles addressing different applications of mHealth technology [38]. These studies spanned the globe and covered all continents except Antarctica and addressed mHealth applications for chronic diseases, transplantation, dermatology, dentistry, health promotion, maternal and child health and so forth [38]. Though many of the included articles had limitations of size, the conclusions of the study were positive for the development of both SMS and application based mHealth solutions for a diverse array of healthcare conditions [38].

Despite challenges and barriers, the prospect of mHealth carries significant potential. According to the WHO’s GOe, the unprecedented spread of mobile and electronic technologies has given over 85% of the world’s population- over 5 billion individuals- coverage with a commercial wireless signal [7]. The development of mobile phone networks in low-and-middle income countries has even superseded infrastructure development of roads, electricity and traditional internet deployment [7]. As technological sophistication continues to advance, speeds of data transmission and availability of lower-cost mobile devices will transform data exchange globally [7]. Since 2011, more progress has been made globally in eHealth. The key findings of the 2016 WHO GOe Report show that 58% of responding WHO Member States have an eHealth strategy, 90% of countries with an eHealth strategy report that they have special funding available for it, 50% of WHO member countries have government-supported health internet sites that offer information in multiple languages and 75% of WHO member countries have institutions that offered training in information communication technologies (ICT) for health professionals [8]. Given these global conditions, it appears that the integration and development of mHealth platforms could potentially bolster capacity, accessibility and delivery of care systems to vulnerable individuals even in the most remotes areas of the world.

We acknowledge our study had limitations. We utilized only four databases (PubMed, PsychInfo, ACM Portal and DOAJ) and therefore we might have missed relevant literature in other repositories. Additionally, our algorithmic search method may have not included literature available outside of the keyword inputs. However, based on the sensitivity of the four databases used for this systematic review and on the appropriate inter-rater reliability of our search methods, evidenced by high Cohen’s kappa values, we are confident that the results of this study are significant. A third limitation of our systematic review lies in the circumstances that prevented the conduction a single meta-analysis - insufficient data availability, lack of qualitative data that would better answer review questions and significant differences in the few identified studies that prevented the combination of data.

Nevertheless, it is exactly this paucity of data in the field of Arab mMHealth technology that exemplifies the current challenges and directions for further research.

## 5. Conclusions

The mental health needs of a massive refugee influx often exceed a host nation’s mental health system capacity to respond adequately. mMHealth technology diffusion and transfer could greatly complement clinic-based care while also bolstering system capacity. A clear understanding of barriers to mMHealth implementation holds the potential of advancing feasible solutions to unexpectedly high demands. The present study affirms the paucity of literature and lack of evidence available regarding the uptake of mMHealth interventions among Syrian refugees and other vulnerable Arab populations. Whether in the Arab Region or in the diaspora of non-Arab host countries, the study also identifies some of the current challenges to mMHealth implementation. Most importantly, the study findings suggest that some the main obstacles to mMHealth usage acceptability in Arab populations, may be the lack of an established theory base congruent with the Arab socio-cultural norms and the lack of a realistic conceptual framing and grounding of the mMHealth uptake problem. Recommendations include a paradigm shift from the current Western models of eHealth acceptance and diffusion, to an evidence-based Arab gendered and culturally competent conceptual framework capable of informing more user-acceptable adaptations of mMHealth.

## Figures and Tables

**Table 1:** PICOS * Outline & Research Contextualization.

Participants	Patients and participants from the Middle East, Arab Culture/Background or Syrian refugees receiving mental healthcare in home or host nations. Healthcare workforce members, community members and all related parties involved in the provision of mental healthcare to the patients and participants outlined in (1) above.
Interventions	Tele-health, mobile Health or electronic health platforms particularly targeted toward mental health provision
Comparisons	Conventional psychotherapy and psychiatric intervention No treatment
Outcomes	Alleviation of mental health burden/symptoms Acceptance of mMHealth technology by study participants Barriers to mMHealth technology
Study Design	Randomized control trials Qualitative studies (Studies that provided data and statistics) Outcomes based studies (Studies that utilized data to discuss differences in outcomes between mMHealth and other treatments)
Research Questions	What is the current state of mMHealth utilization towards Syrian Refugees and Arab populations? What types of mMHealth solutions have been utilized? Are they being utilized for monitoring, reporting, tracking or alleviating mental health burden? Any other uses? If mMHealth applications are being used, what has been their efficacy? Are these solutions effective? What are the barriers to mMHealth utilization in the study populations?

* PICOS = Participants, Interventions, Comparisons, Outcomes, Study Design. Adopted from the PRISMA Guidelines [27].

**Table 2:** PubMed, PsychInfo, ACM and DOAJ Boolean Search Queries.

Journal	Search Query
PubMed	(mobile health OR mHealth OR m-Health OR telemedicine OR tele-medicine OR telepsychiatry OR tele-psychiatry) AND (Syria OR Arab OR Arabic OR Saudi Arabia OR Lebanon OR Jordan OR refugee OR “middle east” OR “Middle eastern”) AND (“mental health” OR depression OR PTSD OR psychiatry OR psychiatric)
PyscInfo	(mobile health OR mHealth OR m-Health OR telemedicine OR tele-medicine OR telepsychiatry OR tele-psychiatry) AND (Syria OR Arab OR Arabic OR Saudi Arabia OR Lebanon OR Jordan OR refugee OR “middle east” OR “Middle eastern”) AND (“mental health” OR depression OR PTSD OR psychiatry OR psychiatric)
ACM	+(“Mobile Health” mHealth telemedicine tele-medicine telepsychiatry tele-psychiatry) +(refugee syria arab arabic “Saudi Arabia’” Lebanon Jordan “Middle East” “middle eastern”) +(“Mental health” depression PTSD psychiatry psychiatric)
DOAJ	(mobile health OR mHealth OR m-Health OR telemedicine OR tele-medicine OR telepsychiatry OR tele-psychiatry) AND (Syria OR Arab OR Arabic OR Saudi Arabia OR Lebanon OR Jordan OR refugee OR “middle east” OR “Middle eastern”) AND (“mental health” OR depression OR PTSD OR psychiatry OR psychiatric)

**Table 3:** Background Information and Key Findings of Included Studies.

Title	Authors	Year Published	Study Type	Objectives	Study Population	Sample Size	Key Findings & Feasibility Results	Barriers Identified
User-Centered App Adaptation of a Low-Intensity E-Mental Health Intervention for Syrian Refugees [28]	Burchert, Alkneme, Bird, et al.	2019	Qualitative study. (Interviews)	To assess mHealth prototype acceptance	Adult Syrian Refugees in Germany, Sweden, and Egypt	128	• 78% reacted positively to the potential health impact of the prototype intervention	• Technical literacy • Problems with internet access • Acceptability • Credibility • Technical requirements
The prevalence and usage of mobile health applications among mental health patients in Saudi Arabia [29]	Atallah, Khalifa, Metwally, et al.	2018	Qualitative study. Cross-Sectional (Surveys)	To explore the prevalence of use of mHealth applications for mental health	Adult, mental health patients in Saudi Arabia	376	• 64% use mobile phones to access health data • 64% expressed interest in using mobile phones to track progress of their mental health • 46% reported running 1-2 healthcare applications on their mobile phones	NA
Community cognitive interviewing to inform local adaptations of an e-mental health in tervention inLebanon [30]	Abi Ramia, Harper-Shehadeh, Kheir, et al.	2018	Qualitative study. (Interviews)	To investigate the use of community-driven adaptation of evidence based e-mental interventions outlined by WHO (Step-by-Step)	Lebanese, Palestinian, and Syrian Health workers and community members in Lebanon	66	• Significant adaptation of WHO’s Step-by-Step online intervention to the Lebanese community • 30% decrease in length • Additional videos with alternate delivery methods • Greater focus on enjoyable activities • Support styles were adapted to gender norms • Several other minor changes	• Gender differences in preferred intervention styles • Length of intervention • Privacy and security of data • General distrust of healthcare providers • Lack of mental health awareness
mHealth for mental health in the Middle East: Need, technology use, and readiness among Palestinians in the West Bank [6]	Ben-Zeev, Fathy, Jonathan, et al.	2017	Qualitative study. Cross-Sectional (Surveys)	To assess mobile phone use and interest in mHealth for mental health	Adult Palestinians in the West Bank	272	• 93.4% own a mobile phone • 99.6% use social media regularly • 88.2% thought mobile health interventions would be helpful to people with mental health problems • 66% report interest in mHealth for mental health	NA
Mental health assessments in refugees and asylum seekers: evaluation of a tablet-assisted screening software [31]	Morina, Ewers, Passardi, et al.	2017	Randomized Crossover Trial	To investigate the efficacy and feasibility of psychological screening software for touch-screen devices	Adult refugees and Asylum seeksers in Zurich, Switzerland originating from Afghanistan, Iraq, Turkey, Sri Lanka, and Sudan.	30	• No significant difference between conventional and mobile application screening, suggesting feasibility and efficacy • Improved time efficiency using mobile application	NA
Facilitating mental health screening of war-torn populations using mobile applications [32]	Hashemi, Ali, Awaad, et al.	2016	Qualitative study. Cross-sectional (Surveys and Interviews)	To investigate the efficacy and utility of a mobile application for mental health screening	Palestinian children in Gaza	986	• Screened children were found to suffer from a wide range of hyperarousal, re-experiencing, depressive, and somatic symptoms • The open data kit (ODK) open source mobile application proved to be an easy, efficient, and feasible data collection tool in a resource constrained setting	NA
Attitudes Towards Implementation of Store-and-Forward Telemental Health in Humanitarian Settings: Survey of Syrian Healthcare Providers [33]	Jefee-Bahloul, Duchen, Barkil-Oteo	2016	Cross-Sectional (Surveys)	To investigate perception and feasibility of use of audio/video recordings for telepsyciatry	Adult Syrian healthcare providers in NGOs working in the Syrian disaster setting	30	• Half of the providers believed that mental healthcare can be provided through audio/video data consultation, and that there would be benefit from such services • Providers generally believed that Syrian patients would agree to be recorded for psychiatric service	• Perceived barriers included cultural, financial, and technical barriers
Web-Based Psychotherapy for Posttraumatic Stress Disorder in War-Traumatized Arab Patients: Randomized Controlled Trial [34]	Knaevelsrud, Brand, Lange, et al.	2015	Randomized Control Trial	To investigate the efficacy of cognitive behavioral internet-based intervention	Adult, War-traumatized Arab patients in Iraq. All participants have PTSD	159 (79 in treatment group)	• 62% of treatment group had recovered from PTSD symptoms at 3-month follow-up versus 1 patient in the control group (*p* < 0.001; OR 74.19)	NA
Pilot Assessment and Survey of Syrian Refugees’ Psychological Stress and Openness to Referral for Telepsychiatry (PASSPORT Study) [35]	Jefee-Bahloul, Moustafa, Shebl, et al.	2014	Cross-Sectional (Surveys)	To investigate psychological burden and openness to telepsychiatry	Adult Syrian Refugees in Kilis, Turkey	354	• 41.8% showed scores indicative of PTSD • 45% willing to use telepsychiatry • 34% reported perceived need to see psychiatrist	The following were negatively associated with willingness to receive telepsychiatry: • Female gender • Bilingual • Positive score testing for PTSD
Transcultural telepsychiatry and its impact on patient satisfaction [36]	Mucic	2009	Cross-Sectional (Surveys)	To investigate patient satisfaction regarding the use of videoconferencing for telepsyciatric care	Adult asylum seekers, refugees, and migrants in Denmark	52	• Patients reported a high level of satisfaction and willingness to use telepsychiatry again and recommend it to others. They preferred telepsychiatry in their mother tongue rather than interpreter-assisted care.	• Limited infrastructure regarding setup of telepsychiatry areas and exam rooms

**Table 4:** Inter-rater Reliability (*K* Table).

Phases of Articles Selection	*k*
**Initial screening**	
1. Author dependence on *Title* for inclusion/exclusion	0.95
2. Author dependence on *Abstract* for inclusion/exclusion	0.87
3. Author dependence on the *Full Article* for inclusion/exclusion	0.83
4. Inclusion of *non-Review* articles (outcomes based studies, qualitative studies and randomized control trials)	0.92
5. Inclusion of *Review* Articles	0.93
6. *Comprehensiveness* of Search	0.92
**Quality Evaluation**	
7. *Quality of Topic*	0.84
8. *Quality of Study Design*	0.95
9. *Scientific Quality of Research*	0.89
